# Correlation between pulp sensibility and magnetic resonance signal intensity following regenerative endodontic procedures in mature necrotic teeth- a retrospective cohort study

**DOI:** 10.1186/s12903-024-04095-y

**Published:** 2024-03-13

**Authors:** Noha Mohamed El-Kateb, Amr Mohamed Abdallah, Rania Noaman ElBackly

**Affiliations:** Conservative Dentistry Department, Endodontics Division, Faculty of Dentistry, Alexandria University, Champollion Street, El Azareta., Alexandra, Egypt

**Keywords:** Endo-Ice test, Electric pulp test, Magnetic resonance imaging, Necrotic pulp, Signal intensity

## Abstract

**Background:**

With increasing studies being published on regenerative endodontic procedures (REPs) as a treatment modality for mature necrotic teeth, the assessment of outcomes following regenerative endodontic procedures has become more challenging and the demand for a better understanding of the regenerated tissues following this treatment is rising. The study aimed to correlate cold, electric pulp testing (EPT), and magnetic resonance imaging (MRI) signal intensity (SI) in mature necrotic teeth treated with regenerative endodontic procedures.

**Methodology:**

This retrospective cohort study included eighteen adult patients who experienced tooth necrosis in mature maxillary anterior teeth recruited from the outpatient clinic, Conservative Dentistry Department, Faculty of Dentistry, Alexandria University, Alexandria, Egypt from July 2017 until December 2018 with 12 months of follow-up. regenerative endodontic procedures via blood clot were performed. The canals were instrumented by ProTaper Next (PTN) files until final sizes X3 or X5. Biodentine was used as cervical plug material. Pre and post-operative clinical follow-up was done where the patients’ responses to cold and electric pulp testing were given a scoring system and were compared to the normal contralateral tooth. Pre and post-operative magnetic resonance imaging signal intensity of both the involved tooth and its contralateral at the middle and the apical thirds of the root canals were assessed after 3, 6, and 12 months. Data was analyzed using the ANOVA, Friedman and Bonferroni tests. Significance was set at a *p*-value < 0.05.

**Results:**

All 18 teeth scored a baseline score of “2” for cold and electric pulp testing. There was a significant difference between scores of the cold test at baseline and 12-month follow-up (*p* < 0.001). There was a significant difference between scores of the electric pulp testing of baseline and 12-month follow-up (*p* < 0.001). There was a moderately significant indirect (inverse) correlation between magnetic resonance imaging signal intensity and cold test in both the middle and apical thirds at 12 months. No significant correlations were detected between magnetic resonance imaging signal intensity and electric pulp testingat any of the time intervals (*p* > 0.05).

**Conclusion:**

Magnetic resonance imaging is a successful non-invasive method to assess outcomes of regenerative endodontic procedures and correlating it with another reliable method of assessing pulpal responses, cold test, could validate these outcomes.

**Clinical trial registration:**

The study was registered with ClinicalTrials.gov (ID: NCT03804450).

## Background

Currently, regenerative endodontic procedures (REPs) are highly recognized for their promising role in the treatment of necrotic mature teeth [1]. They represent a cell-homing-based procedure where different stem cells with regenerative potential are recruited to the tooth via the blood clot [2]. With increasing studies being published on REPs as a treatment modality for mature necrotic teeth [3–13], the assessment of outcomes following REPs has become more challenging. Indeed, the demand for a better understanding of the regenerated tissues following this treatment is rising. The current knowledge of wound healing after REPs is mostly derived from animal /histological studies. Unfortunately, there is still a scarcity of published information regarding the true nature of these tissues on human teeth. Such studies are important because they provide relevant insights regarding the in vivo wound healing outcomes of REPs [14].

Results from the few studies on immature teeth that do exist have shown that while vital tissues appear to be formed within the root canals of these teeth in association with the resolution of infection, these tissues seem more of a repair response rather than a reflection of regeneration of a true dentin-pulp complex [14–16, 3]. One study [4] has shown that the histological outcomes of regenerated tissues in mature revascularized teeth appear to be similar to those for immature teeth with the presence of vascularized fibrous connective tissues with bone-like areas and inflammation.

To fulfill the requirements of successful REPs, the American Association of Endodontists (AAE) guidelines [17] have advised the use of a bacteria-tight coronal seal to be placed over the induced blood clot. Biodentine in endodontics has been designed to be permanently placed in close contact with living tissues not only for its strong antibacterial property [18] but also due to its good biocompatibility [19], sealing ability [20] and its effect on the stem cell proliferation [18]. In addition, it does not cause tooth discolouration [21].

The proprioceptive defense mechanism of the pulp is maintained by regaining tooth sensibility, which provides an alarm system against tissue injury and any further damage. Pulp sensibility tests such as thermal (cold) and electric pulp tester (EPT) are the most used methods to assess the integrity of the Aδ-fibres in the pulp-dentin complex indicating whether there is a neural response from the pulp [22]. However, they do not reflect the true vitality of the tissues [23]. Laser Doppler flowmetry (LDF) and pulse oximetry (PO) can be used to assess pulp perfusion [24] however, the presence of the coronal barrier placed in the pulp chamber following regenerative procedures may prevent accurate measurement.

Pulp sensibility results from many studies have confirmed positive responses which point to the presence of a vital pulp-like tissue [5–12]. We have recently shown that mature necrotic teeth treated with REPs do respond to cold sensibility and electric pulp testing. We further confirmed these results with Magnetic resonance imaging (MRI) where signal intensity (SI) showed the perfusion of the root canals of these treated teeth [13].

Therefore, regaining pulp sensibility and perfusion is an important milestone for regenerative endodontics however, the question of pulp sensibility testing in endodontics is still subject to debate with most techniques falling short of correlating with the actual histopathological status of the pulpal tissues [23]. For REPs, this is even more critical as it is important to have an early measure for the pulpal response, especially with the increasing number of cases that have recently reported the development of significant intracanal calcification following regenerative procedures [3, 25, 26] .

By having a true measure of tissue perfusion, changes that occur with time may reflect the histological development of the tissue response within the canals and hence allow early interference to preclude further complications from happening. Unfortunately, this cannot be achieved by cone beam computed tomography (CBCT) as it cannot visualize soft tissues contained in solid structures [27]. Indeed, the use of MRI may offer a valid non-invasive radiation-free alternative not only for its excellent tissue contrast but also for its ability to visualize dental pulp morphology more clearly [28]. Additionally, changes in the signal intensity in the different locations of the canal and at different time points may provide a real-time assessment of the repair/regeneration process [13].

Therefore, the current study aimed to stratify the patient responses to cold and electric pulp testing using a scoring system upon comparing the responses to the normal contralateral tooth. Furthermore, we aimed to correlate cold, EPT, and MRI SI in revascularized mature necrotic teeth. The null hypothesis of the present study was that there would be no correlation between cold, EPT and MRI SI.

## Methods

### Study design

This retrospective cohort study followed the guidelines for Reporting observational studies in Endodontics. (PROBE, 10.1111/iej.13873). It included eighteen adult participants recruited from the outpatient clinic, Conservative Dentistry Department, Faculty of Dentistry, Alexandria University, Alexandria, Egypt from July 2017 until December 2018.

### Ethical approval

The study protocol was approved by the Institutional Review Board (IRB) of the Faculty of Dentistry, Alexandria University, Alexandria, Egypt, under IRB Number 00010556-IORG 0008839 and dated 22/01/2017. The study was registered with ClinicalTrials.gov (ID: NCT03804450). Written informed consent was obtained from all study participants after the study methodology was explained.

### Sample size

The sample size was estimated assuming 80% study power and 5% alpha error. Based on Iohara et al. [26], the reported mean (SD) MRI SI in regenerated teeth after 180 days = 6.7 (1.1), and in normal teeth = 7.8 (1.0). Based on the comparison of means, the required sample size was calculated to be 16, increased to 18 to make up for procedural problems. The sample size was calculated using G*Power (Version 3.9.1.7).

### Eligibility criteria

The eligibility criteria for the present study were adult male and/or female patients 20–40 years of age, free from systemic diseases and with maxillary anterior necrotic mature teeth with single canals with periapical lesions either due to trauma, caries or defective restoration. Patients with mobile teeth, root fractures, hopeless teeth, teeth with stainless steel wires &/or brackets, pacemakers and implants were excluded from the study.

### Patient pre-operative examination

Medical and dental history was collected from all patients. A preoperative clinical examination was conducted and included: Periodontal pocket depth, sensitivity to percussion and palpation at the apical area of the affected tooth, and sinus tract presence.

Preoperative clinical examination also included cold and EPT. For the cold test examination: A dry cotton roll was placed in the buccal vestibule to isolate the tooth from the lip and cheek. Ethyl chloride spray (Walter Ritter, Hamburg, Germany) was used where a cotton pellet was sprayed resulting in the formation of ice crystals which was then applied to the labial surface of the crown for both the affected tooth and its contralateral with caution not to touch the gingival tissue. Participants were asked to raise their hands when feeling a cold sensation [29]. For the EPT: Parkell Digitest Pulp Vitality Tester (Brentwood, NY) was used according to the manufacturer’s instructions: the tooth was partially isolated and properly dried. A lip clip was used to ensure a complete circuit. A small amount of toothpaste was placed on the tip of the metal probe to ensure contact between the probe and the tooth. The device was activated by pressing and holding the start button for a half second, and then releasing the button, once the display read “00”, this indicated that it was ready to use. The probe was then placed on the middle third of the labial surface of the crown. the button was pressed and held and the rise in the display indicated that a gentle-pulse stimulus was being automatically applied to the tooth. Once a sensation was felt the button was released immediately to stop the flow of the stimulus. This was done on both the affected and contralateral teeth [30].

Preoperative digital radiographs were taken to confirm the presence of a periapical lesion. A preoperative MRI scan was also performed using a 3Tesla (T) machine, using a head and neck coil (Achieva Gyroscan; Philips Healthcare, Best, Netherlands) to measure the SI of both the involved tooth and its contralateral at the middle and the apical thirds of the root canals as previously described [13].

### MRI data acquisition

MRI survey scan was done to build the entire head and neck, axial T2-weighted images, and 3-dimensional (3D) T2-weighted images. The examination included the standard MRI turbo spin echo sequence. The T2 axial parameters were: Repetition time of 54,500 milliseconds, echo time of 100 milliseconds, flip angle of 90, acquisition voxel of 1.3 × 31.3 mm, and 3-mm slice thickness. For the 3D T2 images, the parameters used were: Repetition time of 1500 milliseconds, echo time of 160 milliseconds, acquisition voxel of 1.3 × 31.3 mm, 2.5-mm slice thickness, turbo factor of 40, and a scan time of 3.50 min. The raw MRI data were then collected from the extended workstation (R2.6.3.1, Philips) and analyzed [13]. The region of interest (ROI) for measuring the SI was defined by tracing an elliptical area ranging from 0.01 to 0.02 cm^2^ in the axial slices. This was determined for the middle third of the involved teeth in the axial view immediately apical to the cervical plug material and the corresponding ROI at the contralateral teeth was also measured. The ROI at the apical third for both the involved and contralateral teeth was measured in the axial view immediately coronal to radiographic apex again by tracing an elliptical area ranging from 0.01 to 0.02 cm^2^ in the axial slice at this predetermined level.

### REPs

All the clinical procedures were performed by a single, experienced, investigator (N.M.K) according to the following protocol [13]: At the first appointment, local infiltration with 2% lidocaine (with 1:100,00 epinephrine [Lignospan Standard]; Septodont, Saint-Maur-des-Fosses, France) was done followed by rubber dam isolation and access cavity preparation. The canals were irrigated with 20 mL of 1.5% sodium hypochlorite. After determining the working length and confirming canal patency the canals were instrumented by PTN files until final sizes X3 or X5. Ultracal XS calcium hydroxide (Ultradent Products GmbH, South Jordan, UT) was delivered, 2 mm short of the radiographic apex, according to the manufacturer’s instructions where the teeth were temporized using intermediate restorative material (Dentsply Sirona) until the following appointment. At the second visit, if there were signs/symptoms of persistent infection, the disinfection protocol was repeated. If not, 3% mepivacaine without vasoconstrictor (Scandonest, Septodont) was administered followed by rubber dam isolation. After flushing the calcium hydroxide from the canal using 20 mL 1.5% sodium hypochlorite and applying the final rinse with 20 mL 17% ethylenediaminetetraacetic acid (EDTA) (Prevest Direct, Jammu, India), bleeding was induced into the canals by over-instrumenting by rotating a pre-curved K-file #25 at 2–3 mm beyond the radiographic apex. Biodentine (Septodont) was mixed and carefully placed over the blood clot according to the manufacturer’s instructions followed by light-cured resin-modified glass ionomer restorative material as a base (Riva, SD, Australia) and final composite resin restoration (Nexcomp; Metabiomed, Chungcheongbukdo, South Korea). To prevent traumatic occlusion high points were carefully checked.

### Outcome Assessment

All the postoperative clinical follow-ups were performed by two calibrated investigators (N.M.K & R.N.B). It was done after 1, 3, 6, and 12 months. Scores for the cold and EPT were given according to the following interpretation:

### For the Cold test [29]

The following scores were defined:

- SCORE = 0 for the immediate response to cold that was alleviated within 0–3 s.
- SCORE = 1 for the delayed response with a tingling sensation felt between 4 and 15 s after cold application.
- SCORE = 2 when no response was felt till 15 s.

### For EPT [11, 30, 31]

The following scores were defined:

- SCORE = 0 for immediate response with numerical readings ranging from 0 to 3.
- SCORE = 1 for delayed response with a tingling sensation with numerical readings ranging between 4 and 39.
- SCORE = 2 when no sensation was felt between 40 and 64 (maximum stimulus).

Postoperative radiographic follow-ups were performed by a single, experienced, investigator (N.M.K) and included periapical digital radiographs to assess periapical healing throughout the study. Regarding the MRI SI measurements of the regenerated pulp-like tissue, it was done after 3, 6, and 12 months of follow-up by locating the ROI as previously mentioned by an expert calibrated radiologist.

Calibration on cold and EPT was done for the two investigators (N.M.K & R.N.B). The statistician was blind to the given data.

### Statistical analysis

Data were analyzed using IBM SPSS for Windows (Version 23.0) and significance was set at a *p*-value < 0.05. Comparisons of MRI SI and pulp testing (cold and EPT) at different time intervals within middle and apical thirds were done using repeated measures ANOVA and Friedman tests. This was followed by multiple pairwise comparisons using Bonferroni adjusted significance level to assess the relationship between MRI SI and pulp testing (cold and EPT). In addition, Spearman correlation was used to correlate MRI SI with cold and EPT with the calculation of Spearman correlation coefficient (Rho) and *p* values. The intraclass correlation coefficient (ICC) test was performed to evaluate the intra and inter-examiner reliability.

## Results

**Fig. 1:**
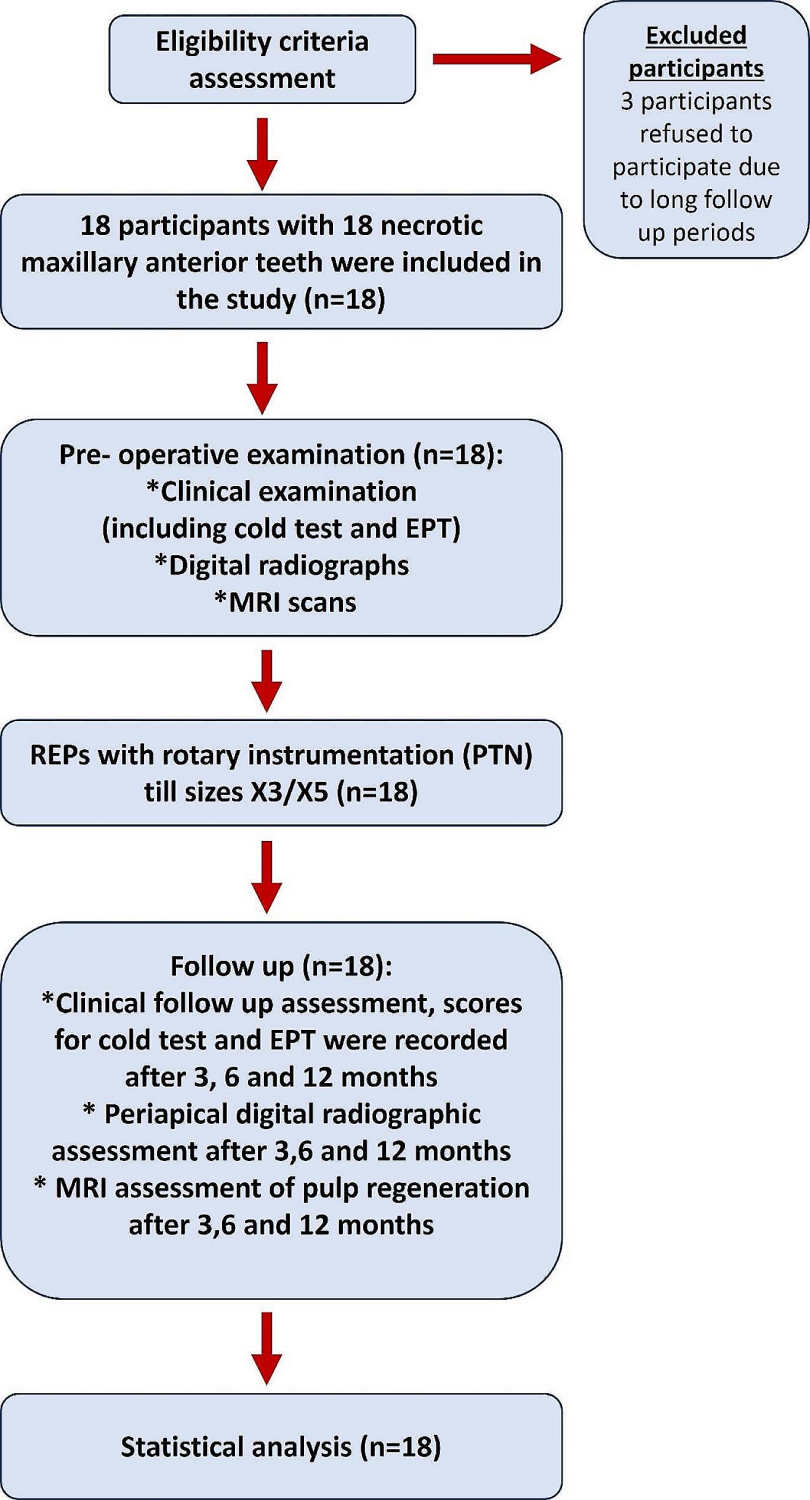
Flow chart of the research design

**Fig. 2:**
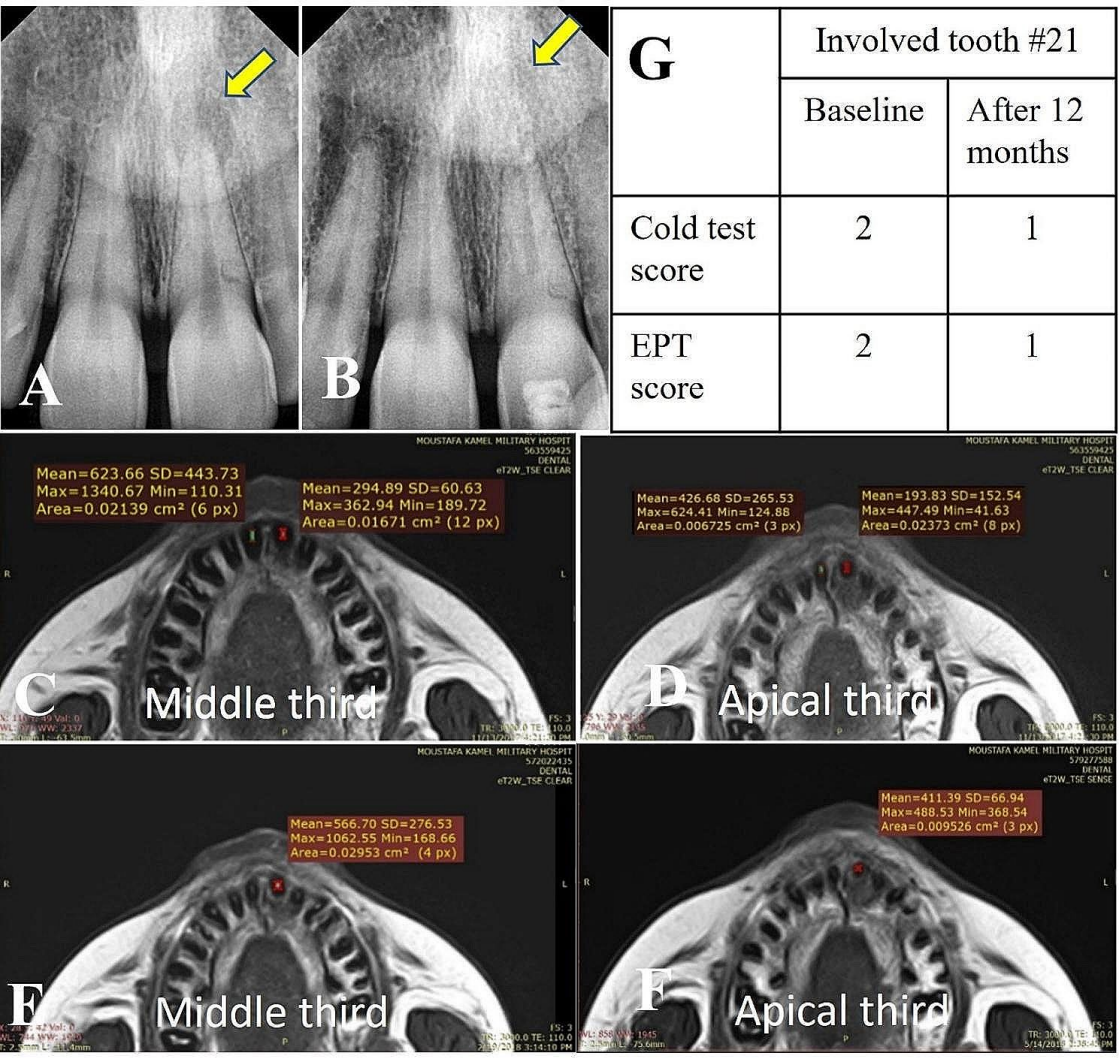
Radiographic images of a representative case: (**A**) Digital preoperative radiographs with periapical lesions related to tooth #21 (yellow arrow). (**B**) After 12 months follow-up showing periapical healing (yellow arrow). (C)(D)(E)(F) MRI signal intensity measurements (**C**) and (**D**) Middle and apical thirds baseline for both involved tooth (#21) and normal contralateral (#11) (**E**) (**F**) Middle and apical thirds after 12 months follow-up period. (**G**) table representing the cold test and EPT scores for the involved tooth #21

**Fig. 3:**
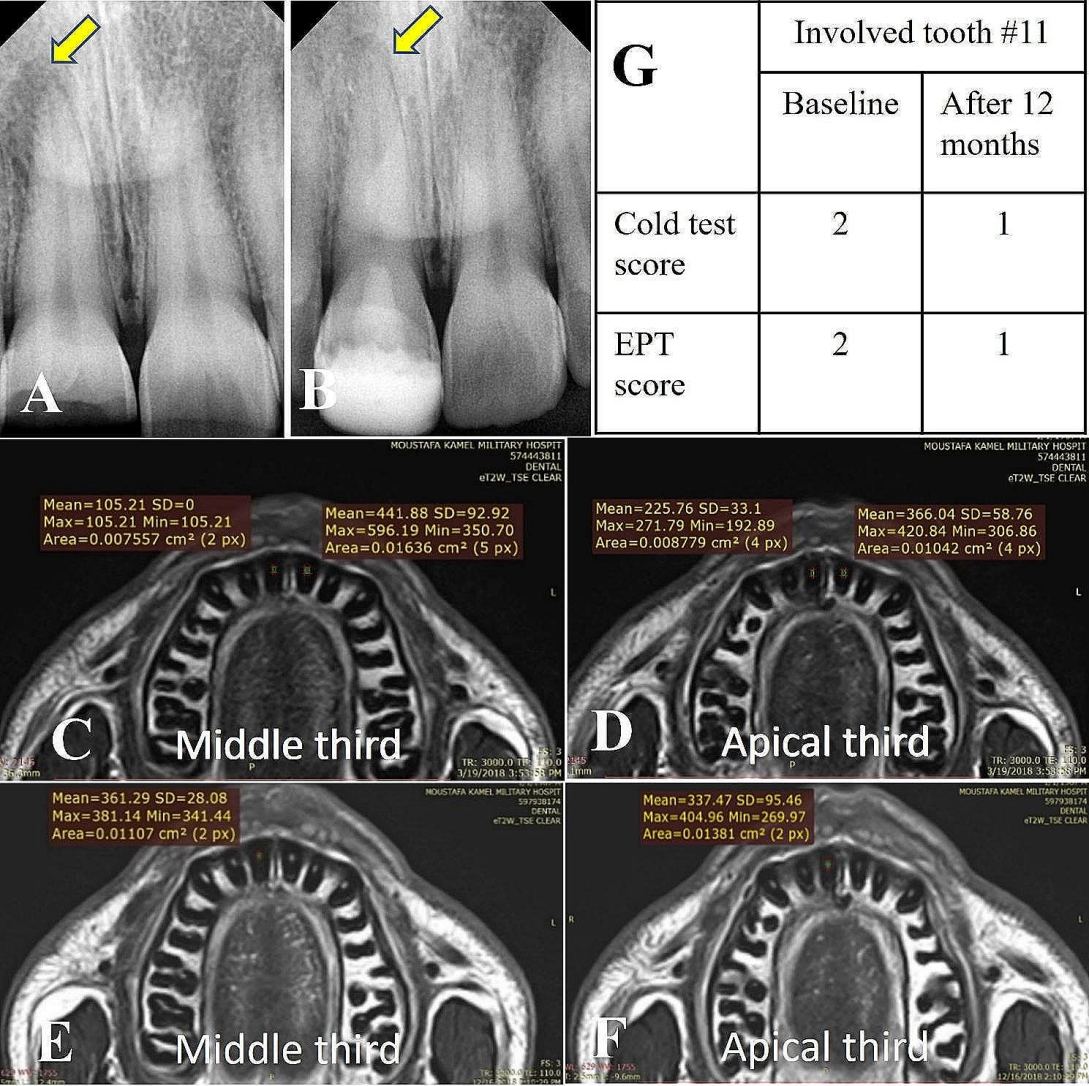
Radiographic images of another representative case: (**A**) Digital preoperative radiographs with periapical lesions related to tooth #11 (yellow arrow). (**B**) After 12 months follow-up showing periapical healing (yellow arrow). (**C**)(**D**)(**E**)(**F**) MRI signal intensity measurements (**C**) and (**D**) Middle and apical thirds baseline for both involved tooth (#11) and normal contralateral (#21) (**E**) (**F**) Middle and apical thirds after 12 months follow-up period. (**G**) table representing the cold test and EPT scores for the involved tooth #11

Twenty-one participants were assessed for eligibility in the present study, 3 were excluded as they refused to participate due to the long follow-up period and 18 participants who met the eligibility criteria were selected for the study (Fig. 1). The clinical examination results showed no pain, no sensitivity to percussion, nor swelling in all 18 teeth throughout the follow-up periods. Periapical healing was enhanced in all cases (Figs. 2 and 3). The ICC ranged from 0.89 to 0.98 indicating excellent reliability between examiners across time.

### Baseline demographics

The baseline demographics and clinical characteristics of the 18 enrolled patients including age, gender, tooth type, etiology of pulp necrosis, and periapical diagnosis are summarized in Table 1.

**Table 1:** Baseline demographics of the 18 patients

Case #	Age	Gender^*^	Affected tooth^$^	Aetiology of pulp necrosis	Tenderness to percussion	Presence of periapical lesion	Presence of sinus tract	Periapical diagnosis
1	34	M	11	Trauma	Absent	Present	Present	Chronic apical abscess
2	20	M	21	Trauma	Absent	Present	Present	Chronic apical abscess
3	32	F	21	Trauma	Absent	Present	Absent	Asymptomatic apical periodontitis
4	21	F	21	Defective restoration	Absent	Present	Absent	Asymptomatic apical periodontitis
5	20	M	12	Trauma	Absent	Present	Present	Chronic apical abscess
6	24	F	21	Trauma	Absent	Present	Absent	Asymptomatic apical periodontitis
7	20	M	21	Defective restoration	Absent	Present	Absent	Asymptomatic apical periodontitis
8	31	F	21	Trauma	Absent	Present	Absent	Asymptomatic apical periodontitis
9	25	F	21	Trauma	Absent	Present	Absent	Asymptomatic apical periodontitis
10	20	M	11	Trauma	Absent	Present	Absent	Asymptomatic apical periodontitis
11	34	F	11	Defective restoration	Absent	Present	Absent	Asymptomatic apical periodontitis
12	27	F	21	Trauma	Absent	Present	Absent	Asymptomatic apical periodontitis
13	20	F	21	Trauma	Absent	Present	Absent	Asymptomatic apical periodontitis
14	21	F	11	Defective Restoration	Absent	Present	Absent	Asymptomatic apical periodontitis
15	32	M	11	Trauma	Absent	Present	Present	Chronic apical abscess
16	33	F	11	Defective restoration	Absent	Present	Absent	Asymptomatic apical periodontitis
17	23	F	21	Trauma	Absent	Present	Absent	Asymptomatic apical periodontitis
18	21	M	21	Trauma	Absent	Present	Absent	Asymptomatic apical periodontitis

^*^Gender: M;Male,F;Female ^$^FDI tooth numbering system

**Fig. 4:**
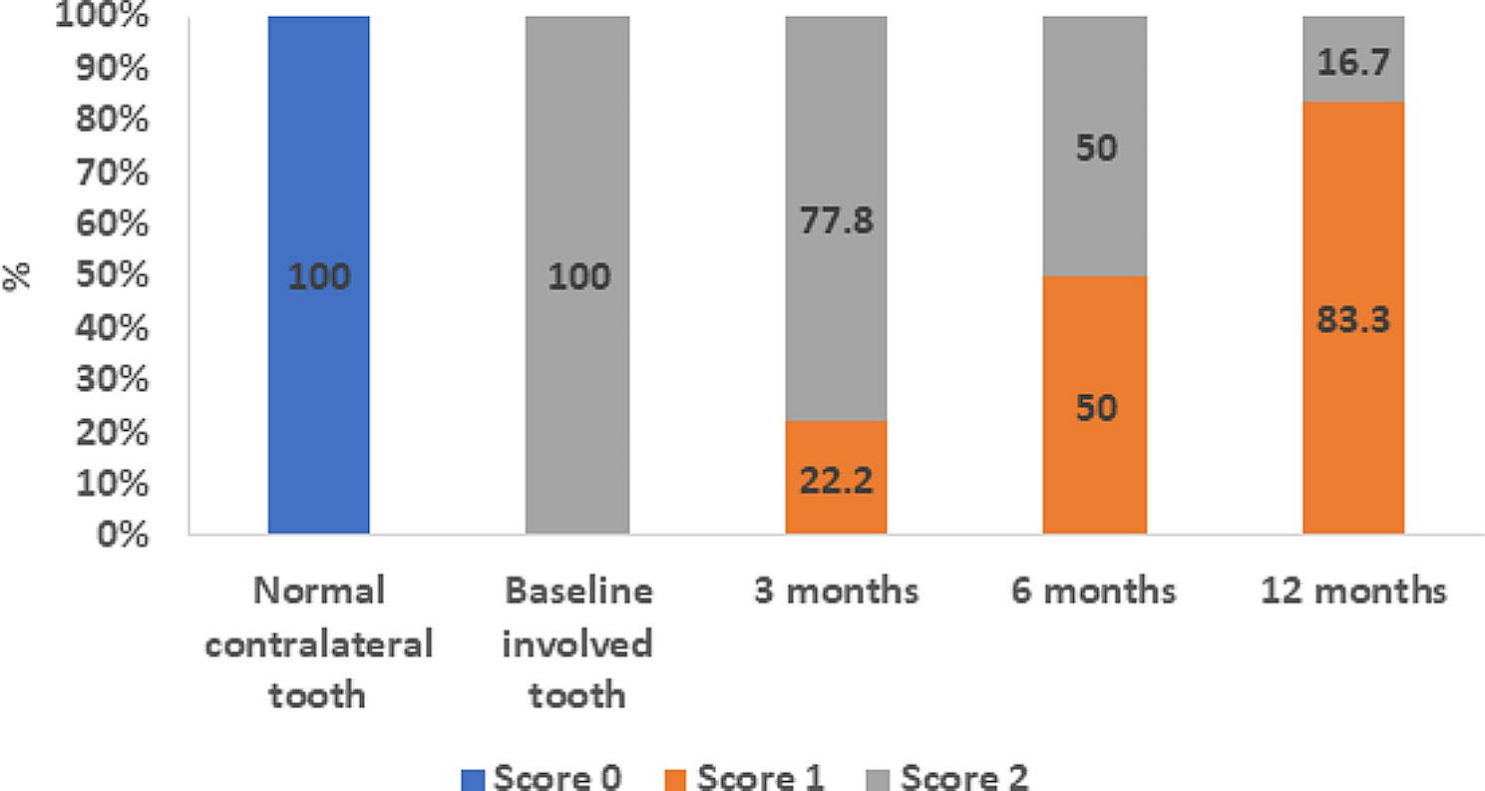
Graph showing the cold test scores at different time points for both normal and involved teeth

**Fig. 5:**
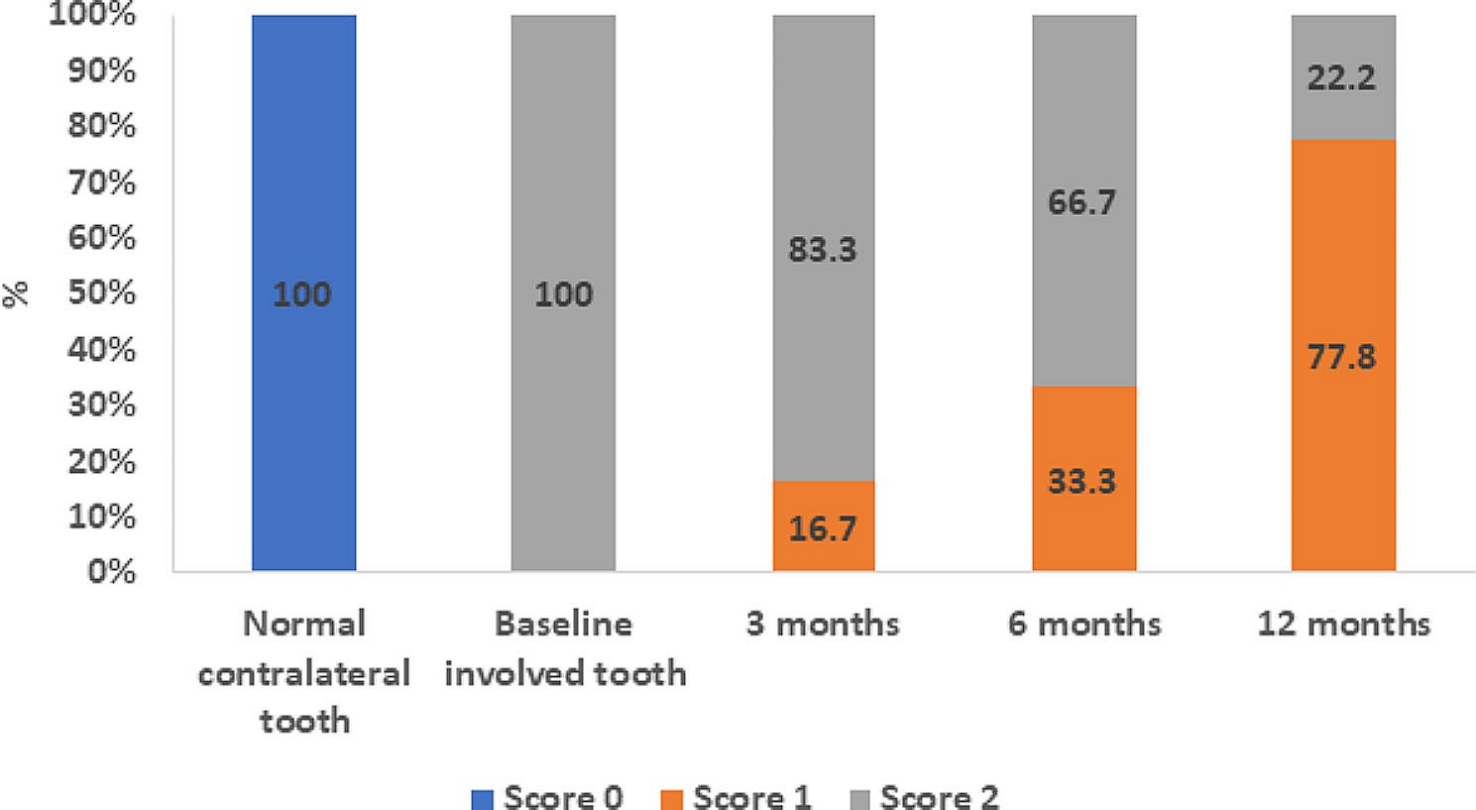
Graph showing the electric pulp test scores at different time points for both normal and involved teeth

For the normal contralateral teeth in all cases, all 18 teeth scored “0” for both cold and EPT indicating healthy pulpal condition (Figs. 4 and 5).

Regarding the cold pulp testing of the involved teeth, at baseline, all teeth recorded a score of “2” (100%), and this decreased to 14 teeth at the 3-month follow-up (77.8%). At the 6-month follow-up, 9 teeth recorded a score of “2” (50%), and the remainder recorded a score of “1”(50%), and at the 12-month follow-up, 15 teeth out of 18 recorded a score of “1”(83.3%) (Fig. 4).

There was a significant difference between scores of the cold test at baseline and those at both 6-month (*p* = 0.02) and 12-month follow-up (*p* < 0.001) and between scores of the 3-month and 12-month follow-up periods (*p* = 0.001) (Table 2). The decrease in the score value indicates regained sensibility.

Similarly, the EPT scored “2” in all 18 involved teeth at baseline. At the 3-month follow-up, 15 of the teeth scored “2” (83.3%), and at the 6-month follow-up 12 of the teeth scored “2” (66.7%) and other teeth scored “1”. At the 12-month follow-up, 14 out of 18 teeth scored “1” (77.8%) (Fig. 5).

There was a significant difference between scores of the EPT of baseline and 12-month follow-up (*p* < 0.001) and between scores of the 3-month and 12-month follow-up periods (*p* = 0.002) (Table 2). Similarly, as with cold testing, the decreasing score value indicates a more rapid regain of pulp sensibility.

**Table 2:** Post hoc multiple pairwise comparisons of cold and EPT at different time intervals in *involved teeth*

Timepoint	Compared to	Cold test *p*-value	EPT *p*-value
**Baseline**	**3 months**	1.00	1.00
	**6 months**	0.02*	0.18
	**12 months**	< 0.001*	< 0.001*
**3 months**	**6 months**	0.57	1.00
	**12 months**	0.001*	0.002*
**6 months**	**12 months**	0.27	0.07

*statistically significant using Bonferroni adjusted significance level

There was a moderately significant direct correlation between MRI SI and cold test scores in the apical third at 3 months (Rho = 0.50, *p* = 0.03) i.e. the higher the SI in the MRI scans, the higher the score value (score 2) i.e. the lower was the response to the cold test. (Table 3). On the other hand, there was a moderately significant indirect (inverse) correlation between MRI SI and cold test scores in both the middle and apical thirds at 12 months (Rho= -0.56, -0.53 for middle and apical thirds, respectively, *p* = 0.02) i.e. the higher the MRI SI at 12 months, the lower the cold score values indicating more regain of pulp sensibility to cold.

No significant correlations were detected between MRI SI and EPT at any of the time intervals (*p* > 0.05) (Table 3).

**Table 3:** Spearman correlation between MRI SI with cold and EPT

		Cold test	EPT
		Rho (*p*-value)	
**Middle third**	**3 months**	Rho = 0.41 *P* = 0.09	Rho = 0.22 *P* = 0.39
	**6 months**	Rho= -0.10 *P* = 0.70	Rho = 0.10 *P* = 0.69
	**12 months**	***Rho= -0.56*** P = 0.02*	Rho= -0.46 *P* = 0.053
**Apical third**	**3 months**	**Rho = 0.50** P = 0.03*	Rho = 0.32 *P* = 0.20
	**6 months**	Rho = 0.10 *P* = 0.70	Rho = 0.36 *P* = 0.14
	**12 months**	***Rho= -0.53*** P = 0.02*	Rho= -0.36 *P* = 0.14

Rho: Spearman correlation coefficient

*statistically significant at *p*-value < 0.05

Although crown discolouration and intracanal calcification were not considered as part of the original study outcome measures, none of the cases showed significant town discolouration after 12 months. Furthermore, while intracanal calcification and root canal narrowing were noticed in the radiographs in several cases, no attempts were made to quantify the extent of calcification observed.

## Discussion

Based on the results of our previously published study [13] and since this is a retrospective cohort study, we chose an apical diameter of 0.3–0.5 mm. Our previous results showed no statistically significant differences in the clinical and radiographic outcomes of cases instrumented either to 0.3 or to 0.5 mm final apical diameter. The selection of this range is based on several findings The first is that the average size of human cells ranges from 10 to 100 μm [32]. Furthermore, Laureys et al. found that 0.3 mm was sufficient to allow tissue ingrowth in an animal mode [33]. Saoud et al. also successfully treated mature maxillary anterior teeth with apical diameter sizes of 30 [6]. On the other hand, a systematic review by Fang et al. [34] concluded that clinical success was achieved after REPs in necrotic teeth with an apical diameter size from 0.5 to 1.0 mm. Moreover, reported studies have successfully performed REPs with an apical diameter of 0.5 mm [6, 32].

A recent common consensus by several studies is that until now the success of REPs in mature necrotic teeth has been mainly focused on periapical healing as a predictable treatment outcome [35]. On the other hand, reporting the restoration of pulp sensibility and/or pulp vitality has been controversial. While some studies have reported positive responses to pulp sensibility and vitality measurement, the results of these studies have shown contrasting values. It is however important to acknowledge that periapical healing as an outcome could mainly be a reflection of adequate disinfection, a goal not to be undermined [36]. Yet, the restoration of functional dental pulp tissue remains the true goal of REPs. Studies in both immature and mature permanent teeth have demonstrated that a mixture of repaired and regenerated tissue can be found in the canals of revascularized/revitalized teeth. Some of these tissues have proven components of vascularized and innervated tissue simulating dental pulp tissue. Although this is a well-desired demonstration of a scientist-centred outcome, it is important to realize that there is a need to correlate between clinical and histological findings and develop a reproducible method to assess the true status of the repaired/regenerated tissues in teeth that have been subjected to REPs. We have previously shown that MRI can be a reproducible, non-invasive method of assessing and quantifying the SI from regenerated tissues following REPs in mature necrotic teeth [13]. However, it is important to correlate these results with routine pulp sensibility tests to gain a better understanding of the nature of these tissues.

Recent concerns regarding the interpretation of the results of pulp sensibility testing of revascularized/revitalized mature teeth have stemmed from the fact that these tests are subjective and mainly rely on the patient’s responses. Additionally, criticism has been directed at these studies due to the absence of testing of sound healthy controls in the same patient. On the contrary, the current study compared SI in regenerated teeth with those of contralateral healthy control teeth giving strength to the results of the correlation outcomes of this study. Additionally, the lack of clear cut-off values in several studies can open the field to bias and increase the subjectivity of the results. This was again addressed in the current study by assigning scores according to the time needed to obtain a reproducible response after both cold and electric pulp testing [1, 37–39]. Regarding the EPT, different studies considered that any sensation felt by EPT represented a vital tooth while no sensation indicated a non-vital tooth [11, 30, 31]. In the present study, we made some modifications to Nageh et al’s [11]methodology by dividing the entire positive response (from 0 to 39) into immediate positive response (0–3 = score 0) and delayed positive response (4–39 = score 1) for better documentation. Previous studies have also attempted to use differences in EPT values to categorize different pulpitis diagnoses [40].

Indeed, the present study aimed to record the patient response following REPs in mature necrotic teeth to cold test and EPT using a scoring system after 3,6 and 12 months of follow-up and to correlate these results with the MRI SI measurements at both the apical and the middle thirds of the canal. In this study, there was a gradual increase in the percentage of teeth regaining tooth sensibility using the cold test and EPT throughout the study periods to reach the highest levels at 12 months (83.3% and 77.8%, respectively).

This was in agreement with the findings of Paryani and Kim [5], Nageh et al [11], and Arslan et al. [12]on mature teeth treated with REPs, indicating the presence of vital tissues. Our results are also similar to Brizuela et al [41] who used a cell-based approach for regenerative endodontic treatment of mature necrotic teeth with periapical lesions. While the results of the current study are slightly higher with regards to regaining pulp sensibility in comparison to the aforementioned studies, they validate the cell-homing approach in its ability to give rise to vascularized innervated and possibly pulp-like tissue. It remains to be seen whether or not the tissues regenerated in Brizuela et al’s study histologically recapitulate dental pulp tissue and whether long-term results would allow the preference for a cell-based versus a cell-homing approach [41]. Positive response to sensibility tests has also been primarily reported in some studies treating necrotic immature permanent teeth with apical periodontitis with REPs [42–44]. Histologically, Lei et al. [45] confirmed neural tissue regeneration by immunohistochemistry in an extracted immature permanent premolar tooth, treated with REPs. This was also confirmed by Arslan et al. on a mature tooth with successful REPs that was extracted due to dental trauma [4].

However, the results of the present study were inconsistent with the Saoud et al. case series [6] and Nagas et al. [10] when mature teeth were treated with REPs. The possible explanation of the nerve regeneration following REPs may be due to several factors such as the blood clot being a rich source of endogenous growth factors [46], the use of 17% EDTA could promote neuronal growth and axonal regeneration as well as cell proliferation, migration, and odontoblastic differentiation [47]. Furthermore, the recruited stem cells in the root canal have been shown to have the potential to differentiate into neuronal cells and can induce axon guidance [48].

Due to the limitation of sensibility tests being subjective and do not assess pulpal perfusion, MRI was used as an objective, quantitative, non-invasive method of assessing the perfusion of regenerated pulp-like tissues in the present study. Because of its excellent tissue contrast and being radiation-free, it was not only able to visualize dental pulp morphology and assess its relation to adjacent anatomical structures, MRI has played an interesting role in the field of regenerative medicine, particularly pulp regeneration, where it has been used to assess regeneration of pulp-like tissue after transplantation of dental pulp stem cells both in vitro and in vivo [49].

The representation of the tooth in an MR sequence depends on the water content in the dental tissues. The pulp tissue which is composed of blood vessels, lymph vessels, connective tissue, nerve tissue, and cells appears bright (due to big water content): hyperintense/high SI. On the contrary, enamel and dentin contain very low water content thus the number of free protons is low, therefore appearing almost black in the MR image: hypointense/ low SI [50]. A recent study using pulpal contrast enhancement of MRI showed that it can represent an objective pulp vitality assessment tool. The study revealed that there were no significant differences in SI in relation to age thus eliminating the influence of age-related changes on pulpal vascularity assessment using this technique. However, the authors did not correlate their results with different levels of response to cold as the study was conducted using only healthy pulps. In contrast, we performed pre- and post-operative analysis and thus we were able to correlate the different tests employed. Furthermore, the use of contrast agents is not always possible and comes with potential unwarranted risks [51].

The MRI SI in our previous study [13] showed a high SI after 3 months of follow-up in all treated teeth indicating the restoration of the vasculature of the regenerated tissue due to angiogenesis compared to the necrotic baseline which lacks perfusion; low water content thus showing low SI. Then the SI decreased gradually throughout the follow-up periods to be nearly equivalent to the SI of the normal contralateral teeth indicating the organization of the regenerated tissue in the canal together with the formation of mineralized tissue and dentin deposition that may decrease the vascularization thus decreasing the SI. This may prove that MRI could be used as an early detection tool for pulp perfusion and could predict the pulpal response to sensibility tests after 6 and 12 months. In 2001, Ploder et al. [52] were able to demonstrate that in transplanted teeth with incomplete root formation showing reperfusion in MRI dental images, positive responses to cold could be elicited after 3–6 months of transplantation. This appears to be a similar phenomenon to that observed in this cohort of revascularized teeth. Another interesting observation they made was that the extent of reperfusion was associated with the development of the roots. This could again correlate with the pattern of SI increase followed by a decrease in the current study; events replicating the formation and maturation of newly regenerated tissues, respectively.

In the current study, there was a significant correlation between MRI signal intensity and the cold test however there was no correlation between MRI signal intensity and EPT. The direct correlation between the cold test scores and MRI signal intensity in the apical third after 3 months may indicate that while earlier deposited tissue may be highly vascularized (high SI), it might lack the maturation of nerve fibres sufficient to produce a rapid response to cold testing hence registering a higher cold test score or delayed response. There was no significant correlation however with the SI in the middle third after 3 months. It is important to note that while MRI can differentiate the SI of the tissue at different levels, the cold test would reflect the response of the tissue anywhere within the revascularized root canal.

This could also reflect the process of tissue maturation. It has been previously shown that in revascularized teeth, tissue in-growth through the apical foramen may occur to different levels; sometimes reaching up to fill only the apical third, in other cases reaching the middle third or even coming close in contact with the coronal plug material. Tissues found in the apical third are also often associated with substantial amounts of hard tissue depositing on the canal dentinal walls. Contrarily, tissues may not have matured substantially in the middle third of the canal to elicit a valid response to cold at that time point [53–55].

On the other hand, as the tissues mature with time more homogenously throughout the canal and their signal intensity approaches that of normal tissues, they may become more mature in their innervation hence stimulating a more rapid detectable response to cold after 12 months or in other words lower cold test scores. These results may further highlight the vascular and neural events that take place during the regeneration of new vital intracanal tissue. This could confirm the results from previous diagnostic studies that concluded that the cold test represents the most sensitive and reliable method of assessing the pulpal status compared to EPT [56]. Furthermore, it has also been shown that while negative EPT readings are quite reliable, low positive values can indicate a tendency for false-positive readings [57, 58]. Noteworthily, response times to cold testing have been shown to increase in elderly patients [58]. The authors explain that this could be due to the presence of secondary and sclerotic dentin. Likewise, progressive canal calcification and hard tissue deposition following REPs may result in decreased SI with time as well as delayed responses to cold as compared to the normal contralateral teeth.

In a recent study, Mittal et al. [59] assessed the pulp sensibility following REPs in mature teeth using different scaffolds. They concluded that teeth in all groups with different scaffolds gained a positive response to the cold test after 12 months and none gained a positive response to EPT. They suggested that this could be due to the high sensitivity and reliability of the cold test compared to EPT and the coronal plug materials prevent the conduction of the electric current. This could further support why there was no correlation between the MRI and the EPT. It has also been shown that the re-establishment of vitality responses would require targeting periapical neuronal networks into coronal areas of the regenerated tissues 10–15 mm away from the nearest nerve trunks. This may be hindered by the depth of apical extension of the coronal plug material preventing transmission of stimulus and hence detecting a pulpal response to EPT [60].

On the other hand, the response to cold may be due to the direct detection of temperature by dental afferent neurons which are known to express thermosensitive ion channels which are sensitive to temperatures below 25ºC. This possibly explains that even though the higher percentage of cases registered a score of “1” (delayed response) at earlier time points, this temperature change was sufficient to be detected by neurons in the newly regenerated tissues. Additionally, after 12 months, the increased response could indicate a more mature tissue as well as a more coronal ingrowth of newly regenerated tissues. A further possibility to explain the response to cold is the release of adenosine triphosphate (ATP) from neighbouring cells and their detection by purinergic receptors [58]. Indeed, this can further explain the strong correlation between the MRI signal intensity and the cold test scores in the different canal thirds.

While the results of this study are promising, there are some limitations. One of these is the inability to correlate the findings with the histological assessment of regenerated tissues as it is invasive and requires tooth extraction. Another limitation is that MRI remains an expensive tool limited to hospitals, and needs qualified trained technicians hence it is currently not possible to recommend it as a routine method of analysis following REPs. Another limitation is that the image quality could be negatively affected by any metallic appliances and patient minor movements such as breathing, swallowing and anxiety. Other limitations included difficulty in inducing blood in teeth with closed apices together with the difficulty in accessing the level of Biodentin due to its similar opacity as dentine. Finally, the inability to accurately compare the results of the study to other randomized clinical trials due to their scarcity and the heterogeneity of the methods used.

In conclusion, the results of the current study further corroborate our previous findings that regenerative endodontic procedures in mature permanent teeth can give rise to regenerated tissues functionally analogous to dental pulp tissue. Furthermore, we confirm that cold sensibility testing can be a reliable measure to assess pulpal responses following REPs however, due to the technical uniqueness of the REP model, responses cannot be expected to be identical to normal healthy pulps early following treatment and long-term follow up is needed to truly judge the success of treatment. Measuring MRI SI and correlating it to cold test results can provide more validation of the outcome of REPs. However, longer-term and multi-centre clinical trials are still needed to reach a predictable measure of success regarding the regain of pulp sensibility following REPs in mature necrotic teeth. This would in turn allow the development of a precise core outcome [61] set to aid in clinical decision-making for applying REPs in the treatment of mature teeth.

## Data Availability

The datasets used and/or analysed during the current study are available from the corresponding author upon reasonable request.

## Abbreviations

3D: 3-dimensional
ATP: Adenosine triphosphate
AAE: American Association of Endodontists
CBCT: Cone Beam Computed Tomography
EPT: Electric pulp testing
EDTA: Ethylenediaminetetraacetic acid
IRB: Institutional Review Board
ICC: Intraclass Correlation Coefficient
LDF: Laser Doppler flowmetry
MRI: Magnetic resonance imaging
PTN: ProTaper Next
PO: Pulse oximetry
REPs: Regenerative endodontic procedures
ROI: Region of interest
SI: Signal intensity
PROBE: Reporting observational studies in Endodontics
T: Tesla
